# Fracture healing in India: Available therapies, indications, and protocols

**DOI:** 10.4103/0019-5413.50853

**Published:** 2009

**Authors:** Michel Saccone, Anil K Jain

**Affiliations:** Division of Orthopaedic Surgery, Department of Surgery and Department of Clinical Epidemiology and Biostatistics, McMaster University, Hamilton, Ontario, Canada; Division of Orthopaedic Surgery, University College of Medical Sciences and GTB Hospital, University of Delhi, New Delhi, India

**Keywords:** Fracture healing, bone stimulation, India, indications, protocols, available therapies

## Abstract

The availability of fracture healing therapies to the general public is limited in India. The infrastructure of the health system in India, involving both public and private sectors, does not provide adequate opportunity for rural and low-income inhabitants to access needed care. Also the lack of funding from the government and the overall lack of physicians place a large strain on the system. This paper will take an in-depth look at the state of the current health care system and how it affects bone stimulation therapy in India. The Indian Journal of Orthopaedics was used as a reference for the bone stimulation therapies currently utilized in India. A general search of the therapies and technologies was performed to determine protocols and indications. A table of fracture healing therapies and technologies was composed which provides a description of each therapy, as well as its specific indications and protocols. This information was then used by the authors to hypothesize the most feasible methods of fracture healing to meet the Indian demographic. Based on an assessment of the health system of India, the most practical methods of bone stimulation therapy were determined. It was also determined that nearly all forms of therapy could be made available if sufficient resources were set aside for it. Bone stimulation therapy in India remains a large void in the health care system.

## INTRODUCTION

India's National Health Policy (NHP) was first developed in 1983, indicating the inexperience of and lack of development in its National Health System compared to other nations of the world. The NHP was based on the premise of providing access to health for all.^1^ However, being an underdeveloped nation, India was stricken with a high prevalence of infectious diseases. The Ministry of Health and Family Welfare (MHFW) had to set its priorities to control the abundance of infectious diseases and improve the living conditions for all.^1^ After the rate of infectious disease declined, India could focus on attempting to attain the reputation of a developed nation. To do this, India required a number of improvements in its health system.

In 2002, India released its second version of the NHP.^2^ The new NHP made notable progression towards the development of a reputable and effective health system; however, many deficiencies remain. The MHFW upgraded their goal of providing “access to health for all” to providing an “acceptable standard of health to the general population” by 2015.^2^ The main postulates of the second NHP were to increase the unacceptable funding currently provided by the government to the public health system, to improve health care equality across all states, and to continue attacking infectious diseases with specific, goal-driven expectations.^3^ In 2005, India inhabited nearly one-fifth of the world's population and possessed over a fifth of the world's diseases. The demand on the health system was and continues to be overwhelming.^3^ Obtaining sufficient funding and controlling population growth are imperative to maintaining advancements in health. So far, policy changes have been nullified by the massive population growth.^2^ The inability to control issues resulting from poor nutrition and hygiene have drained the health system of resources.^1^ India will need to utilize its health resources to the fullest potential.

India is passing through a major sociodemographic, epidemiologal, technological, and media transition. Health scenario has also altered. India had 58,863,000 vehicles in 2002, 197 times greater than 1951, with 71% two wheelers.^4^ The unprecedented increase in vehicles without road safety norms has lead to tremendous increase in road traffic accidents. The national average of deaths due to road traffic injuries is about 800/lac of population.^5^

The projected deaths in India because of road traffic accidents were 850,000 for the year 2005^4^. The ratio of death to serious injuries needing hospitalization to minor injuries was reported as approximately 1:20:50. In 2005, approximately 850,000 deaths, 170,000,000 serious injuries needing hospitalization, and 425,000,000 minor injuries were anticipated.^4^ The patients who suffered from serious injuries and minor injuries mainly have fractures of one or more bones. Studies on domestic injuries do not exist in India.

India has sufficient urban-based health services while 72% population lives in rural areas (550,000 villages).^6^ Orthopedic surgeons are mainly concentrated in metropolitan cities. Approximately 70,000 traditional bone setters treat 60% of trauma patients.^6^ The availability of orthopedic surgeons and operation theatre facilities is scarce in rural areas. When these fractures reach the metropolitan city, they already either have a malunion, nonunion, or an established infected nonunion following an open fracture.^6^ The open reduction and internal fixation performed with the use of substandard implants in suboptimal operation theatre conditions produce an infected nonunion.^6^ There is no uniform standard of care available throughout the country and even in the same geographical areas. Hence, besides fresh fractures, the hospitals treat a tremendous number of nonunions, malunions, and infected nonunions.^6^ Besides providing stability at the fracture site, the surgeon needs to augment the osteogenic potential which is acting suboptimally in delayed unions, nonunions, and infected nonunions.

Although no hospital-specific data are available, on an average day, any big hospital treats 75 fresh fractures cases, out of which at least 5–8 require operative intervention.^6^ Almost 500–700 cases of nonunions and infected nonunions are treated per year.^6^ Each metropolitan city has a number of such hospitals hence India has to deal with an enormous burden of fresh fractures and complicated trauma.

In 1985, India yielded the world's highest number of motor vehicle disabilities with 5.4 million occurring annually.^7^ This, coupled with the multitude of disabilities produced from poor working conditions with heavy and unsafe machinery in India's production facilities, places a large stress on the economy. The availability of specialized surgeons and necessary medical equipment and drugs is crucial to the treatment of these injuries.^5^ In particular, fracture healing therapies such as ultrasound, electrical stimulation, and extracorporeal shock wave therapy (ESWT) may help patients to regain health. Stimulating agents and processes such as bone morphogenetic protein (BMP) and biphasic calcium phosphate (BCP) among others are needed to maximize chances for a full posttrauma recovery.

The following discussion will provide insight into the presence of fracture healing therapies and technologies utilized in India. It will describe the protocols and indications of these therapies, as well as determine the effects of the current health system in India on access to these therapies.

## MATERIALS AND METHODS

### Search strategy

It was determined that the indications and protocols of the prominent fracture healing therapies and technologies found in India would be researched. Once it was completed, an in-depth search for information on the Indian health system was conducted. This information would prove useful in hypothesizing which fracture healing therapies and technologies are most applicable to the economy and demographics of India. Articles were located using PubMed, Google Scholar, and the Indian Journal of Orthopaedics database with a broad search strategy.

### Eligibility criteria

The authors defined the eligibility criteria for general fracture healing therapy articles as follows: (1) the article must be peer-reviewed; (2) the article must be written in English; (3) the article must discuss the protocols or indications of the therapy; and (4) the article must have a source in India, as this would indicate that the therapy discussed in the article is likely used in India. Also all applicable articles located in the Indian Journal of Orthopaedics were used to determine major fracture healing therapies utilized in India.

For obtaining information on the Indian health system, the following eligibility criteria were utilized: (1) nonpublished data from the Indian government website; (2) nonpublished information provided by the World Health Organization; and (3) Google Scholar-derived surveys on the Indian Health System.

### Exclusion criteria

Articles were excluded if they did not provide additional insight into the protocols and indications of fracture healing therapies. Primary articles utilized were from the Indian Journal of Orthopaedics or had a source in India indicating the likelihood for the use of the therapy in India.

## RESULTS

The protocols and indications of common and present fracture healing technologies and therapies are summarized in Tables 1 and 2, respectively. However, these stimulations may or may not be found in India. There were no data available on the current or past therapies used in India.

**Table 1:** Fracture healing technologies

Fracture healing therapy	Description	Indications	Protocols
Ultrasound therapy	Low-intensity ultrasound waves are used to stimulate bone growth.^8^ The mechanism involves an increase in the number of neurotransmitter receptors at the fracture site.^9^	It is used in situations of delayed unions and nonunions of bone fractures.^9^ It is also used in fresh fractures to stimulate fracture healing.^9^	Ultrasound machine transducer is used in direct contact with the skin with an ultrasonic gel over the area of the fracture site.^8^ Duration of treatment lasts for 10.20 min.^8^
Extracorporeal shock wave therapy	Pressure waves that tend to have high positive pressures with short wavelengths are used.^10^ The waves travel through fluids and soft tissues until they reach the bone–soft tissue interface where the pressure waves create compression, yielding osteogenesis.^10^ The exact mechanism of bone stimulation is still not fully understood.^10^ Effectiveness is extremely dose dependent.^10^	It is used in situations of delayed unions and nonunions of bone fractures.^10^	Head of the shock wave device is placed on the skin over the fracture site; an ultrasonic gel is used.^11^ Depending on the bone, different levels of shock waves are applied.^11^
Electrical stimulation herapy	Electrodes are placed in the bone and produce a direct, localized stimulation.^12^ It provides a constant direct current, rather than pulsed pressure waves.^12^	It is used in nonunions in large bones such as the femur and tibia and pseudoarthrosis.^12^	A single or multiple electrodes are inserted into the bone via drilled holes. Holes are made in an oblique manner.^12^ The entire cathode is insulated except for the tip which allows for the localized stimulation in the fracture site.^12^ The source of the current is an externally placed anode.^12^ It can be done during initial operation or postoperatively if a nonunion occurs.^12^

**Table 2:** Fracture healing therapies

Fracture healing therapy	Description	Indications	Protocols
Bone morphogenetic protein (BMP)	It is a member of the transforming growth factor (TGF) family; the proteins are naturally produced in the body.^13^ It is dimer protein synthesized by osteoblasts.^14^ It has a large role in the stimulation of extracellular matrix formation.^14^	It is used in delayed unions and nonunions, as well as open fractures.^14^	BMP (usually in a paste or microsphere state) is added to a carrier material that provides a matrix (maintains the volume of the space where bone growth will occur) and is able to maintain the concentration of BMP at the fracture site.^14^
			Carrier types include collagen; inorganic materials such as ceramics; and synthetic polymers such as glass.^14^
Biphasic calcium phosphate (BCP)	BCP is a calcium phosphate compound that acts as a scaffold for bone to grow in and around.^16^ It is a combination of tricalcium phosphate (TCP) and hydroxyapatite (HA).^16^ The design is to provide a structure and a mineral source, yet be able to be degraded by the body so that bone can grow in.^16^ Current evidence shows that HA provides a more stable scaffold, while TCP is more easily degradable.^16^	It is used to treat bone defects and nonunions.^16^	HA and TCP are combined to create the BCP.^16^ BCP is usually delivered in a ceramic form.^16^ It is placed in the gap of the bone (defect), much in the same manner as BMP is utilized.^16^
Parathyroid hormone (PTH)	PTH is an anabolic hormone that is used to increase bone formation by increasing the efficiency of osteoblasts.^17^	It is been approved by the FDA to treat postmenopausal women suffering from osteoporosis, who are at a high risk of fractures.^17^	It can be utilized through subcutaneous injections.^18^
Tissue engineering and gene therapy	Stem (mesenchymal) cells or other cells are used to increase the differentiation by *in vitro* introduction to the carrier material.^19^ The differentiation of cells within the scaffold tends to be even, so when introduced to the fracture site it avoids the issue of cells migrating to areas with greater nutrient sources as the new cells bond tightly with those that are already present and begin bone formation.^20^	It is used in fresh fractures and nonunions.^19^ It is very time consuming.^19^	Stem cells are removed from the patient via a biopsy and developed *in vitro* into the desired type of cell (usually osteoblasts).^19^ The cells are then introduced into a ceramic- or other scaffold-like compound where they are allowed to differentiate.^19^
Calcium sulfate	It has a very rapid resorption rate and for the most part is used as a composite with other bonestimulating agents such as HA or BMP.^21^ It fills the bone void creating space for growth, while it also prevents alternate tissue in-growth.^10^ As the calcium sulfate is absorbed, it demineralizes the bone, stimulating osteogenesis.^13^	It is used to repair any bone defects including those in the long bone, the cranium, and the spine.^21^	Composites of calcium sulfate are entered into the bone voids.^21^ It can also be used successfully in combination with an autogenous bone graft.^13^
Coralline	It is traditionally developed with a form of calcium phosphate, such as HA.^22^ It basically indicates that the implant is very porous and spongelike, and hence is osteoconductive.^22^	It is used to repair any bone defects.^22^	Coralline and calcium phosphate are implanted at the fracture site.^22^ Can be utilized along with an autogenous bone graft, as it has been shown to produce increased bone growth.^22^
Type I collagen	It is the scaffold carrier material that is introduced to the fracture site as a composite.^22^ It is usually in combination with compounds such as HA and TCP.^22^	Used in segmental bone defects and most prominently in long-bone defects.^22^	In its clinically available form, Type I collagen is mixed with 5% Type III collagen, and microparticles are formed.^22^ These particles are then mixed with a ceramic of BCP and placed into the void of a bone.^22^
Allografts or demineralized bone matrix (DBM)	DBM provides the mineral components required for bone growth.^23^ Allografts combine the osteoinductive DBM with osteoconductive agents, such as cancellous allogenic chips, to maximize bone stimulation.^23^	Used in fresh fractures and nonunions.^23^	Internal fixation must occur, which is then followed by grafting.^23^ Grafting is the introduction of the DBM and osteoconductive agents to fill the fracture void. The DBM and cancellous allogenic chips are mixed prior to the introduction.^23^

## DISCUSSION

### The health system

India's current health system is a multileveled infrastructure that thrives on intersectional cooperation for its success. At the national level, there is the MHFW.^1^ The MHFW develops the NHP and is the source of all national programs and changes in the health system.^1^ It is the indirect source of funding for the health system, because of its direct contact with the national government. The MHFH also has a subdivision, which monitors the Indian System of Medicine and Homeopathy (ISMH): cultural medicinal practices that have remained an integral part of the Indian health system. At the state level, there is the Department of Health and Family Welfare (DHFW), which provides the opportunity to direct resources to specific areas of need. Each State DHFW is informed by regional and district level councils and organizations, so that the exact needs of the population are met to the greatest extent.

As for the clinical infrastructure, Indian health care is based on both public and private sectors.^2^ The public sector uses a three-tier system in an attempt to provide necessary care to the rural areas of the country.^1^ The system uses Health Sub-Centers (HSCs) as its basic rural care. Each HSC has available qualified health workers and are located so that there is one for every 5,000 people.^1^ The second tier involves Primary Health Centers (PHCs), in which one doctor is present along with a staff of health workers. Each PHC is designated to 30,000 persons. The third tier is a Community Health Centre (CHC). There is one CHC for every 100,000 people. Despite the large population to accommodate for, each CHC has only 30 beds available and limited selections of specialized doctors.^1^ For most of the rural population, especially for those who cannot afford private care, this is the extent of the health care currently made available by the Indian government. In urban cities, private hospitals account for 68% of the hospitals nationwide.^1^ Public health care is also present in the form of Urban Health Centers (UHCs) and General Hospitals.^1^ UHCs tend to have similar specifications as CHCs, while General Hospitals tend to handle any patient overflow from UHCs.^3^ This clinical infrastructure is clearly unable to meet the needs of the enormous Indian population. With regard to the number of allopathic physicians available in both the public and private sectors, there are 7 per 10,000 persons.^1^ That number pales in comparison to the United States of America where they have 16 per 10,000.^1^ If all private doctors are removed from the statistic, there are only 2 available for every 10,000 people.^1^ Similarly, there are 8 nurses available for every 10,000 people, whereas the global average is 33.^1^ These numbers do not include the nonallopathic physicians of the ISMH as they would be unable to aid in medical emergencies.^1^ The effect of a lack of physicians is compounded in rural communities. It results in numerous vacancies at the second and third tiers of the health system. There are simply not enough incentives for physicians to endure the tumultuous environment that rural India presents. PHC and CHC physicians are required to treat a broad range of illnesses and injuries in a very high volume, with minimal resources and equipment.^7^ It forces doctors to treat patients functionally rather than effectively, which doctors are not traditionally taught to do.^4^ Since it is a profession that is highly regarded, practicing medicine in these sub-standard conditions is not desirable.

The notable lack of funding from the government for public health is a serious barrier to providing proper and effective health care. Based on the 2002 NHP, the government only spent 5.1% of the GDP on health care in 2001.^2^ That expenditure is divided between multiple government ministries.^3^ The MHFW only accounts for roughly 1% of the GDP provided.^3^ The absence of an identifiable source of funding makes it difficult to hold one sector of the government accountable. The NHP has set a goal to spend 8.5% of the GDP expenditure on the health system by 2011.^10^ Despite this, the public health system remains underfunded, and since it provides health care for over a quarter of the Indian population living below the poverty line, the NHP has not provided sufficient funding to provide care to the entire Indian population.^3^

### Availability of fracture healing treatments

The current Indian health system poses a risk to the availability of fracture healing treatments across the country. For middle- and upper-class families located in the urban regions of the country, access to the necessary treatments is not an issue through the use of the private health care system.^3^ However, this is not the case for a large portion of the population.

Fracture healing therapies can be quite expensive when the cost of equipment, materials, training, and research and development is accounted for. The cost to provide and maintain these therapies in Indian practice relies heavily on the functionality of the treatment. If the treatment is not resource efficient, it may not be utilized due to the high demand and low-resource environment that India exhibits, despite being more effective. Fortunately, the cost for diagnosis and treatment in India is markedly lower than that in other parts of the world.^3^ In fact, as shown in Table 3, treatments can be found to cost five times less than in the United States of America and twice as inexpensive as the same treatment in Thailand.^3^

**Table 3:** Comparison of costs for advanced surgery in India, Thailand, and USA^3^

	Cost for treatment in each Country* (US $)		
	
Treatment	India	Thailand	USA
Bone marrow transplant	30,000	62,500	250,000
Open heart surgery (CABG)	5,000–7,000	14,250	30,000
Hip replacement	4,500	6,900	–
Knee surgery	4,500	6,900	20,000
Hysterectomy	500	2,000	–

Gall bladder removal	555	1,755	–

* Cost of surgery in specialized hospitals (private fospitals in India). Source: www.ibef.org

Availability to fracture healing therapies is largely dependent on the availability of physicians to provide surgical procedures and treatments. In the private health system, patient needs can be met for the most part because of the number of doctors available and resources present.^3^ Since all private hospitals are found in urban settings and are purely focused on curative care, the private system provides the best opportunity to receive needed treatment.^3^ Despite this, 85% of private hospitals have less than 10 beds, and specialized hospitals account for only 2% of the total number of hospitals.^3^ This clearly indicates a focus on outpatient treatment, which severely downplays the roles of internal fixation of bone defects. Without the ability to provide overnight stays for patients, many fracture healing therapies and technologies are unable to be utilized, unless they cannot be avoided. An even more staggering fact is that these small hospitals actually account for roughly half of all inpatient hospitalizations in India.^3^

The rural regions of India undoubtedly provide the poorest opportunity to receive fracture healing therapies. The infrastructure currently in place simply cannot meet the requirements of the citizens. With a single allopathic physician for every 30,000 people, not only will the demand of the doctor likely be so high that he or she will be unable to provide adequate fracture healing therapy, but also the resources, if already low in urban centers, will be nonexistent rurally. Proximity of these health services will also likely play a key role. If the injury or illness is not deemed life threatening, it is likely that individuals will turn to the more easily available ISMH, and live with any disabilities that may result from the lack of proper care.

### Fracture healing in India

The “gold standard” of fracture healing therapies has been and is currently the autogenous bone graft.^13^,^15^ The procedure, despite being very effective in bone unification after a significant fracture, is fraught with disadvantages [Table 4].^13^ In fact, roughly 30% of bone grafts have resulted in complications.^13^,^20^,^23^ Consequently, research in this field is now focused on developing an alternative treatment to the autogenous bone graft that can maintain its effectiveness, minimize its disadvantages, and be affordable for the world's population [Table 5].^13^ Affordability is the most important factor for fracture healing in India. Since the public health system receives minimal funding, most, if not all, of it is spent on resources deemed as necessary.

**Table 4:** Disadvantages of an autogenous bone graft10

Limited availability
Postoperative pain at the operative site
Potential injury to the lateral femoral cutaneous
Potential injury to superior gluteal artery
Postoperative hematoma
Potential for infection at the operative site
Possibility of the gait disturbance

**Table 5:** Features of an ideal bone graft substitute10

Have results as good as or better than autograft in achieving union
Be cost effective
Have no immunogenicity
Have handling characteristics familiar to surgeons
Resorb with a predictable degradation time
Act locally without any or negligible systemic side effects
Be osteoconductive and osteoinductive with a potential of supplying
or attracting osteogenic cells
Not interfere with modern imaging modalities
Produce nonexothermic reaction when implanted so as to prevent
heat damage to antibiotics and growth factors

Due to the lack of health statistics data produced by the government of India, it is difficult to determine the exact extent of fracture healing therapy use in India. However, based on the resources available on the Indian Journal of Orthopaedics database, prominent fracture healing therapies were determined. The use of biophysical stimulation known as pulsed electromagnetic fields (PEMFs) is very common.^25^,^26^,^28^ PEMF has been shown to be effective in fresh fractures, osteotomies, and nonunions.^25^ Ultrasound stimulation is also commonly used.^25^ Another form of noninvasive bone stimulation that is used is ESWT.^26^,^28^ As for invasive treatments, the autogenous bone graft is common, as well as allografts, osteochondral grafts, muscle-pedicle bone graft, nonvascularized graft and free vascularized grafts from the iliac or fibular bone.^27^,^28^ All have been shown effective; however, the vascularized graft is preferred to the others as it has a greater success rate of bone stimulation because of its increased angiogenesis.^27^,^28^ When there is a large amount of graft required, bovine calcium hydrocyapatite is utilized, as well as BMP.^28^,^29^ The use of these treatments in the public health system is dependent on the funding present and the skill of the physician as some of the procedures are difficult. Based on the information gathered, it is assumed that these bone stimulation therapies are not used very often outside of urban centers. The lack of use of fracture healing therapies in India does not reflect the need of or the interest in this technology; it simply reflects a lack of initiative to bring such innovations to the Indian marketplace with an economically viable model. Surgeons in India want to improve the healing times in their patients, and patients in turn, are willing to spend money on technologies that work. Ultimately, identifying the optimal fracture healing technology for the Indian population is of significant interest to the health care community.

The private health system is at least one portal to provide an opportunity to adopt novel fracture healing therapies. The actual choice of which therapy is used is currently based on physicians' preference rather than high-quality evidence from randomized trials. The 2007 National Family Health Survey displayed that private health care is utilized by 70% of urban residents and 63% of rural residents.^24^ Given the massive expansion of the middle class in India, private health care has become readily more affordable to millions and millions of Indians who want an access to novel technologies.

### Summary

Health care in India has developed significantly in the past 30 years, yet many voids still remain. The largest void is proper funding for the public health system, as the current government funding is not adequate enough to provide needed care to the entire population. The availability of novel fracture healing therapies in India is difficult to determine and it is largely assumed that the private health care system provides the greatest access to leading fracture healing adjuncts such as growth factors, proteins, and external fracture healing devices. Research must be conducted to further assess the presence and effectiveness of fracture healing therapies in India.
